# Observational study of the effects of age, diabetes mellitus, cirrhosis and chronic kidney disease on sublingual microvascular flow

**DOI:** 10.1186/2047-0525-2-7

**Published:** 2013-04-09

**Authors:** Toby Reynolds, Amanda Vivian-Smith, Shaman Jhanji, Rupert M Pearse

**Affiliations:** 1 Adult Critical Care Unit, Royal London Hospital, Barts Health NHS Trust, Whitechapel Rd, London E1 1BB, UK; 2 Intensive Care Unit, Royal Marsden Hospital, Fulham Road, London SW3 6JJ, UK; 3 Barts and The London School of Medicine and Dentistry, Queen Mary’s University of London, Turner Street, London E1 2AD, UK

**Keywords:** Microvascular flow, Sidestream dark field imaging, Age, Diabetes mellitus, Cirrhosis, Chronic kidney disease

## Abstract

**Background:**

Sidestream dark field (SDF) imaging has been used to demonstrate microcirculatory abnormalities in a variety of critical illnesses. The microcirculation is also affected by advancing age and chronic comorbidities. However, the effect of these conditions on SDF microcirculatory parameters has not been well described.

**Methods:**

SDF images were obtained from five groups of 20 participants: healthy volunteers under the age of 25, healthy volunteers over the age of 55, and clinic patients over the age of 55 with one of diabetes mellitus, cirrhosis and stage 5 chronic kidney disease. Microcirculatory parameters between the groups were then compared for significance using analysis of variance for parametric and the Kruskal-Wallis test for non-parametric data.

**Results:**

Median microvascular flow index was 2.85 (interquartile range 2.75 to 3.0) for participants aged <25, 2.81 (2.66 to 2.97) for those aged >55, 2.88 (2.75 to 3.0) for those with diabetes mellitus, 3.0 (2.83 to 3.0) for those with cirrhosis and 3.0 (2.78 to 3.0) for those with chronic kidney disease (*P* for difference between groups = 0.14). Similarly, there were no significant differences in the proportion of perfused vessels and perfused vessel density between the groups.

**Conclusions:**

Older age, diabetes, and chronic kidney and liver disease need not be considered confounding factors for comparison of SDF microcirculatory parameters in the critically ill.

## Background

Critical illness is often accompanied by abnormalities of the microcirculation [1]. Persistent alterations such as heterogeneity of flow and microvascular shunting can lead to tissue hypoxia and are associated with organ failure and death [2-4], while impaired microvascular flow in the perioperative period is associated with the development of complications [5]. Improving systemic measures of perfusion can be accompanied by improved microcirculatory parameters [6] but the relationship between the two is not always straightforward [7,8], including during vasopressor treatment [9,10]. Monitoring the microcirculation may thus help evaluate an individual patient’s tissue perfusion [7,11].

The novel techniques of orthogonal polarisation spectral (OPS) imaging [12], and more recently sidestream dark field (SDF) imaging [13] allow imaging of the microcirculation *in vivo* in a way that was previously technically difficult. Research using these techniques has helped characterise the sublingual microcirculation in various acute diseases, and describe the changes in microcirculatory parameters following acute physiological changes or administration of pharmacological therapies. However, the influence of existing chronic conditions on microcirculatory assessments made using SDF and OPS imaging has not been well studied. Advancing age, diabetes mellitus, cirrhosis, and end-stage renal failure are commonly found in critically ill perioperative patients [14,15]. All have well recognised associations with an abnormal microvasculature, and altered microvascular flow has been demonstrated using other techniques [16-19]. In order to interpret current and future clinical studies of the microcirculation using SDF and OPS imaging, it is essential to determine whether stable patients with advanced age and these chronic diseases exhibit changes in sublingual microvasculature when assessed using these techniques. The objective of this study was to help provide this understanding by evaluating sublingual microvascular flow using SDF imaging in healthy older volunteers and in patients with diabetes, cirrhosis and chronic kidney disease (CKD).

## Methods

### Study design

This was a single centre, observational study based at a university hospital, and was approved by East London and the City Research Ethics Committee (ref. 09/H0704/3). Patients aged over 55 years with one of either diabetes (diagnosed >2 years), cirrhosis (confirmed by liver biopsy or expert hepatology opinion) or CKD (stage 5 by estimated glomerular filtration rate, but not on dialysis) were identified from amongst those attending diabetology, hepatology or nephrology outpatient clinics. Healthy volunteers aged either under 25 or over 55 years without medical disease were identified from among hospital staff and their relatives. All participants gave written informed consent. Exclusion criteria were pregnancy, age 18 years and refusal of consent.

### Data collection

Sublingual microvascular flow was evaluated using sidestream dark field imaging with a x5 objective lens (Microscan, Microvision Medical, Amsterdam, Netherlands) [20]. Image acquisition and subsequent blinded analysis was performed according to published consensus criteria [21]. SDF images were obtained from at least three sublingual areas. Microvascular flow index (MFI) was calculated after dividing each image into four equal quadrants. Quantification of flow was determined using an ordinal scale (0: no flow, 1: intermittent flow, 2: sluggish flow, 3: normal flow) for small (<20 μm) and large (>20 μm) vessels. MFI is the average score of all quadrants for a given category of vessel size. Vessel density was calculated by inserting a grid of three equidistant horizontal and three equidistant vertical lines over the image. Vessel density is equal to the number of vessels crossing these lines divided by their total length. Flow was then categorised as present, intermittent or absent to calculate the proportion of perfused vessels (PPV) and thus the perfused vessel density (PVD), an estimate of functional capillary density (FCD). Analysis of the videos was performed by a single, blinded observer (TR). Additional data collected for all participants included age, weight, gender, past medical history, current medications, pulse rate, sublingual temperature, heart rate and blood pressure. In diabetic patients the most recent HbA1c percentage result was recorded. In patients with cirrhosis the current Child-Turcotte-Pugh score was recorded. In patients with renal disease, the creatinine clearance was estimated from plasma creatinine using the four-variable modified diet in renal disease method [22].

### Statistical analysis

Data from a previous study, performed locally, demonstrated a difference in MFI of 0.3 prior to major abdominal surgery in those patients who went on to develop complications after surgery compared to those who did not [5]. Assuming a type I error rate of 5% (two-tailed) and a type II error rate of 10% using this previous data, 20 patients would be required in each group to detect a difference in MFI of 0.26 (SD ± 0.25). We therefore aimed to recruit 20 participants in each group. Parametric data are presented as mean (standard deviation, SD) and non-parametric data are presented as median (interquartile range, IQR). Variable distributions were assessed for normality using the Kolmogorov-Smirnov test. The primary outcome measure of this study was a difference in small vessel MFI between the groups. Secondary outcome measures were differences in PPV and PVD for small vessels. Differences between groups were tested using analysis of variance (ANOVA) for parametric data and the Kruskal-Wallis test for non-parametric data. Significance was set at *P* <0.05. Statistical calculations were performed in SPSS (SPSS Inc, Chicago, IL, USA).

## Results

One hundred participants were recruited between September 2010 and July 2011. Adequate video footage was obtained in 98 participants (20 aged <25 years, 20 aged >55 years, 20 with diabetes, 18 with cirrhosis, and 20 with stage 5 CKD). Participant characteristics and baseline clinical data are displayed in Table 1. All the healthy young participants had no medical problems, were non-smokers and declared no regular medication use. Two healthy older participants took medication for hypothyroidism (normal thyroid function tests), two were smokers, two took statins and two took antihypertensive medication, all for primary prevention of cardiovascular disease. All diabetic patients suffered from type 2 diabetes, nine took insulin and 15 took antihypertensive medication. The mean glycated haemoglobin (HbA1c) for patients with diabetes was 8.8% (SD 1.7%). For participants with cirrhosis, three took propranolol for variceal bleeding prophylaxis, and eight took other antihypertensive medication. The cause of cirrhosis was viral in 11 cases, alcohol-related in five cases, medication-related in one case and cryptogenic in one case. Seventeen patients were Child-Pugh-Turcotte score A and one was score B. For patients with stage 5 CKD, this was due to intrinsic renal disease in seven cases, polycystic kidney disease in one case, reflux nephropathy in one case, drug toxicity in one case, and hypertension in ten cases. All were taking antihypertensive medication. The mean estimated glomerular filtration rate was 11.5 ml/min (SD 2.9).

**Table 1:** Baseline participant data

	**Age <25 years**	**Age >55 years**	**Diabetes mellitus**	**Cirrhosis**	**Chronic kidney disease**
Male	10 (50%)	9 (45%)	10 (50%)	12 (67%)	14 (70)
Age (years)	24 (23–25)	61 (60–65)	64 (62–72)	60 (57–66)	71 (64–74)
Body mass index (kg/m2)	21.9 (20.9–24.9)	24.7 (23.0–29.0)	29.4 (24.8–32.9)	24.8 (23.7–26.9)	28.0 (24.6–33.3)
Sublingual temperature (°C)	36.5 (36.3–37.0)	36.4 (36.0–36.7)	36.2 (36.0–36.5)	36.6 (36.2–37.2)	36.5 (36.5–36.8)
Mean arterial pressure (mmHg)	93 (91–96)	100 (93–109)	92 (88–102)	98 (89–106)	98 (89–106)
Heart rate (bpm)	72 (63–82)	75 (65–83)	72 (62–86)	74 (64–92)	70 (65–80)

Data presented as n (%) or median (IQR). n = 20 for all groups except cirrhosis where n = 18.

**Figure 1:**
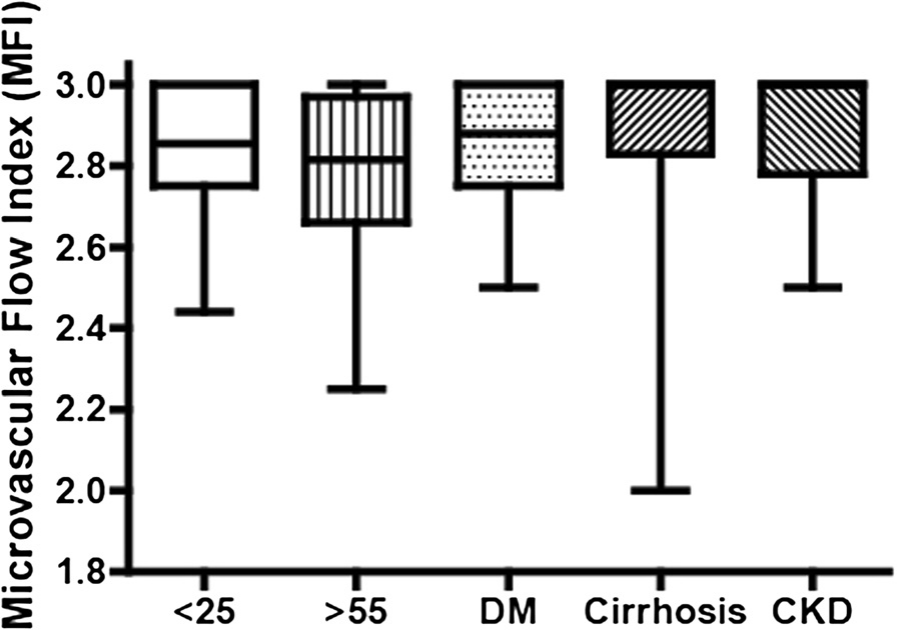
**Microvascular flow index for all participants.** Microvascular flow index (MFI). Data presented as median (IQR). n = 20 for all groups except cirrhosis where n = 18.

A median of 3 (IQR 2 to 4) clips were analysed for each patient. For small vessels (<20 μm), there were no statistically significant differences in MFI, PPV and PVD between the groups (Figure 1 and Table 2). This finding was repeated in a secondary analysis that excluded the two hypertensive participants from the healthy >55 group. Baseline sublingual large vessel (>20 μm) MFI was median 3.0 (IQR 2.9 to 3.0) and PPV was median 1.0 (IQR 0.97 to 1.0), suggesting good quality image capture unaffected by pressure artefact.

**Table 2:** SDF microvascular flow parameters for all participants

	**Age <25 years**	**Age >55 years**	**Diabetes mellitus**	**Cirrhosis**	**Chronic kidney disease**	***P *** **value**
MFI	2.85 (2.75–3.0)	2.81 (2.66–2.97)	2.88 (2.75–3.0)	3.0 (2.83–3.0)	3.0 (2.78–3.0)	0.14
PPV	0.92 (0.06)	0.88 (0.09)	0.92 (0.07)	0.89 (0.08)	0.91 (0.05)	0.46
PVD	8.04 (1.49)	8.02 (0.96)	9.17 (1.70)	8.51 (1.43)	8.38 (1.36)	0.08

PPV in %, PVD in mm^-1^. Data presented as mean (SD) or median (IQR). n = 20 for all groups except cirrhosis where n = 18. MFI, microvascular flow index; PPV, proportion of perfused vessels; PVD, perfused vessel density.

## Discussion

The principal finding of this study was that sublingual SDF imaging does not demonstrate statistically significant differences in MFI, PPV and PVD between healthy young volunteers, healthy older volunteers, and patients with diabetes, cirrhosis and end-stage renal failure. MFI was lower in older volunteers and higher in those with cirrhosis and renal failure, and PVD was higher in patients with diabetes, cirrhosis and renal failure. However, none of these trends reached significance.

There is little doubt that age, diabetes, cirrhosis and renal disease do cause alterations to the microvasculature. Diabetes, particularly type 2 diabetes, is associated with increased capillary density. This has been recently demonstrated using SDF imaging of the labial microcirculation [23]. Our results showed a trend towards an increased PVD that was not statistically significant. Similarly, our results indicated a non-significant trend towards higher PVD and MFI values in cirrhotic and CKD patients. In cirrhosis, vasodilatory mediators promote arteriovenous shunting, eventually leading to the development of a hyperdynamic circulation. Cirrhotic patients show an exaggerated post-ischaemic hyperaemia response, which has been interpreted as evidence that the peripheral microvasculature is predisposed to vasodilatation [24]. Sheikh and colleagues recorded seemingly low MFI values in their comparison of SDF analysis of compensated, decompensated and septic cirrhotic patients [25], but as their study did not include any healthy patients, comparison with our own results is not possible. Using nailfold microscopy, Thang and colleagues found a trend towards reduced capillary density in CKD stage 5 patients compared to controls, although this did not reach significance [26]. Bemelmans and colleagues compared MFI in dialysis patients before and after dialysis [27]. The baseline MFI values they found are similar to those we report, but without a control group in their study, comparison is not possible.

We found that microvascular flow as measured by SDF parameters was not significantly different when subjects were older or had comorbid diabetes, chronic kidney disease or cirrhosis. It is possible that a much larger study than ours may have identified differences between these groups and young, healthy subjects using SDF parameters. It is also possible that alternative techniques may have detected differences that were not apparent using SDF imaging. However, our aim was to identify important confounding effects in the use of SDF parameters to assess for acute changes in the microcirculation of critically ill patients. Assessment of the microcirculation specifically using SDF imaging has generated significant research interest in perioperative medicine and critical care, in part because this technique can be used *in vivo* with minimal disruption to the subject and could potentially form an important part of bedside clinical evaluation. In calculating our sample size, we specified an effect size equal to a 0.26 difference in MFI, on the basis that effect sizes of this magnitude have been seen in previous studies of critically ill patients. Thus we believe our results show that no such confounding effects need be taken into account.

We cannot rule out the possibility that pre-existing microvascular dysfunction not apparent on SDF imaging may predispose patients with chronic diseases to SDF-detectable microvascular flow alterations when they become acutely unwell. Further research would be needed to investigate the importance of any such effect.

## Conclusions

Advancing age, diabetes mellitus, chronic kidney disease and cirrhosis do not appear to cause changes in sublingual microvascular flow assessed by sidestream dark field imaging in stable patients.

## Abbreviations

CKD: chronic kidney disease; FCD: functional capillary density; IQR: interquartile range; MFI: microvascular flow index; OPS: orthogonal polarisation spectral; PPV: proportion of perfused vessels; PVD: perfused vessel density; SD: standard deviation; SDF: sidestream dark field.

## Competing interests

The authors declare they have no competing interests.

## Authors’ contributions

TR, AVS, SJ and RP contributed to the study conception and design. TR and AVS recruited participants, recorded and analysed the data. TR and SJ performed the statistical analysis. TR, AVS, SJ and RP contributed to interpretation of the data and drafting the manuscript. All authors read and approved the final manuscript.

## Authors’ information

RP is a National Institute for Health Research (UK) Clinician Scientist.
